# Application of Ti-in-zircon thermometry to granite studies: problems and possible solutions

**DOI:** 10.1007/s00410-019-1585-3

**Published:** 2019-05-27

**Authors:** David Schiller, Fritz Finger

**Affiliations:** Department Chemistry and Physics of Materials, University of Salzburg, Jakob-Haringer-Strasse 2a, 5020 Salzburg, Austria

**Keywords:** Ti-in-zircon thermometry, Granite petrology, TiO_2_ activity, Zircon saturation

## Abstract

The application of the Ti-in-zircon thermometer to granitic rock requires consideration of $a_{{{\text{TiO}}_{2} }}$ and $a_{{{\text{SiO}}_{2} }}$ during zircon crystallization. Thermodynamic software programs such as rhyolite-MELTS or Perple_X permit the estimation of $a_{{{\text{TiO}}_{2} }}$ and $a_{{{\text{SiO}}_{2} }}$ values from whole-rock geochemical data as a function of pressure and temperature. Model calculations carried out on a set of 14 different granite types at 2 kbar, 5 kbar, and H_2_O = 3 wt% show $a_{{{\text{SiO}}_{2} }}$ during zircon crystallization close to 1 (0.75–1) and $a_{{{\text{TiO}}_{2} }}$ generally far below unity (0.1–0.6). This would suggest that Ti-in-zircon temperatures for granites must be significantly upward corrected relative to the original TiO_2_- and SiO_2_-saturated calibration of the thermometer. Both the rhyolite-MELTS and Perple_X calculations indicate that $a_{{{\text{TiO}}_{2} }}$ is typically around 0.5 in ilmenite-bearing granites. Thus, for ilmenite-series granites (that is, almost all S-type and many I-type granites), it could be a reasonable first order approximation to apply a constant temperature correction of + 70 °C to the Ti-in-zircon thermometer. Granites lacking the paragenesis zircon–ilmenite, that is, some A-type granites and a few special I-type granites may have even lower $a_{{{\text{TiO}}_{2} }}$ (0.1–0.5) and some of them may require a huge upward correction of Ti-in-zircon temperatures on the order of 100–200 °C. Using a set of Ti-in-zircon measurements from a Variscan granite of the Bohemian Massif, we introduce a novel *T*-dependent $a_{{{\text{TiO}}_{2} }}$ and $a_{{{\text{SiO}}_{2} }}$ correction of Ti-in-zircon calculated temperatures which is based on $a_{{{\text{TiO}}_{2} }}$-, $a_{{{\text{SiO}}_{2} }}$–*T* functions modelled with rhyolite-MELTS. This method takes into account that early and late zircons in granitic systems may crystallize at different $a_{{{\text{SiO}}_{2} }}$ and $a_{{{\text{TiO}}_{2} }}$. Furthermore, we highlight the usefulness of comparing the corrected results of Ti-in-zircon thermometry with bulk-rock-Zr-based zircon solubility thermometry and ideal zircon crystallization temperature distributions for granites, and we present a graphical method that enables this comparison. In addition, this paper addresses the problem that Ti-in-zircon measurements are commonly collected with only moderate spatial analytical resolution, which leads to an averaging effect and to difficulties in recording accurate crystallization temperatures. Therefore, we propose that Ti-in-zircon thermometry for granites should generally rely on the more representative median-*T* (*T*_med_) value of a series of zircon analyses. Peak magma temperatures will be, in general, 35–50 °C above *T*_med_, as can be modelled using zircon crystallization temperature distributions.

## Introduction

The Ti-in-zircon thermometer (Watson et al. 2006) could become a most interesting petrological tool for research on granitic rocks if we succeed to overcome a few ongoing problems. The thermometer is based on the temperature-dependent exchange reaction:

$${\text{ZrSiO}}_{ 4} \, + \,{\text{TiO}}_{ 2} \, = \,{\text{ZrTiO}}_{ 4} \, + \,{\text{SiO}}_{ 2} \left( {\text{A}} \right).$$

A phase equilibrium investigation of the system “zircon + rutile + silicate melt/hydrothermal solution” at 1 GPa (Ferry and Watson 2007) gives the amount of Ti in zircon as a function of temperature as:

1 $$\log \left( {{\text{ppm}}\;{\text{Ti}}} \right) = 5.711 \pm 0.072 - \frac{{4800\left( { \pm \;86} \right)}}{{T\left( {\text{K}} \right)}}.$$

While the thermometer is ideally suited for rocks that carry cogenetic zircon, rutile, and quartz (e.g., high-pressure granulites), its application to granites is not straightforward. First, most granites lack rutile implying that $a_{{{\text{TiO}}_{2} }}$ must have been below unity when the zircons formed. Second, although granites are SiO_2_-rich, $a_{{{\text{SiO}}_{2} }}$ can be below unity during early zircon crystallization, because quartz is normally not present at near liquidus conditions (Johannes and Holtz 1991). An equation that corrects the Ti-in-zircon thermometer for the effects of reduced $a_{{{\text{TiO}}_{2} }}$ and $a_{{{\text{SiO}}_{2} }}$ is given by Ferry and Watson (2007):

2 $$\log \left( {{\text{ppm}}\;{\text{Ti}}} \right) = 5.711 \pm 0.072 - \frac{{4800\left( { \pm 86} \right)}}{{T\left( {\text{K}} \right)}} - \log \left( {a_{{{\text{SiO}}_{2} }} } \right) + \log \left( {a_{{{\text{TiO}}_{2} }} } \right).$$

Initially, Ferry and Watson (2007) supposed $a_{{{\text{TiO}}_{2} }}$ to be typically between 0.6 and 0.9 and only rarely below 0.5 in rocks lacking rutile at magmatic temperatures. Further investigations have documented that volcanic rocks with $a_{{{\text{TiO}}_{2} }}$ ~ 0.5 may be quite common (Hayden and Watson 2007; Vazquez et al. 2009; Reid et al. 2011; Ghiorso and Gualda 2012), but systematic study on the variation of $a_{{{\text{SiO}}_{2} }}$ and $a_{{{\text{TiO}}_{2} }}$ in magmatic rocks has not been done so far. We will present such a study in this paper, using the thermodynamic software programs rhyolite-MELTS (Gualda et al. 2012) and Perple_X (Conolly and Petrini 2002). Our results clearly suggest that Ti-in-zircon temperatures for granites have to be significantly upward corrected relative to the original TiO_2_- and SiO_2_-saturated calibration of the thermometer, due to generally low $a_{{{\text{TiO}}_{2} }}$. In addition, we emphasize that zircon crystallization in a granite typically takes place over a larger temperature interval of 80–100 °C (Ickert et al. 2011) and that $a_{{{\text{SiO}}_{2} }}$ and $a_{{{\text{TiO}}_{2} }}$ can potentially change when the magma temperature falls. Therefore, we introduce a new method for a *T*-dependent $a_{{{\text{SiO}}_{2} }}$ and $a_{{{\text{TiO}}_{2} }}$ correction of Ti-in-zircon thermometry data.

During this study, we realized that it is highly advantageous to combine Ti-in-zircon thermometry with Zr-solubility calculations (Watson and Harrison 1983) and we present a diagram that allows both datasets to be effectively compared. Inconsistency between results from the two thermometers indicates that analytical or methodological complications (Siegel et al. 2018) are at play. Furthermore, we highlight a few additional methodological problems, which often arise when Ti-in-zircon thermometry is applied to granites, and we discuss possible solutions.

## Methodological approach

### Thermodynamic modeling

Rhyolite-MELTS (Gualda et al. 2012) and Perple_X (Conolly and Petrini 2002) are thermodynamic calculation software which work by minimization of the Gibb’s free energy for a given bulk rock, pressure, and temperature. The stable phases, their amounts (as percentage), and specific compositions are then calculated for a given *P*–*T* range. Both software programs require whole-rock geochemistry data as an input. Care must be taken that Fe_2_O_3_ and FeO are independently determined. Mineral buffers can be used instead of fixed Fe^2+^/Fe^3+^ ratios if there are concerns that the Fe^2+^/Fe^3+^ ratios received alteration at subsolidus conditions. According to our experience, the C–COH and QFM buffers give good results for ilmenite-series granites (Ishihara 1977) and for magnetite-series granites, respectively.

We used a water content of 3 wt% for all our calculations, as this is considered a realistic value for many granitic magmas (Clemens and Vielzeuf 1987). We also found that varying the amount of water did not significantly change the calculated activities.

Neither rhyolite-MELTS nor Perple_X provide $a_{{{\text{TiO}}_{2} }}$ and $a_{{{\text{SiO}}_{2} }}$ values directly. These must be further calculated from affinity and chemical potential values output from rhyolite-MELTS and Perple_X, respectively. According to Ghiorso and Gualda (2012), the TiO_2_ affinity value ($a_{{{\text{TiO}}_{2} }}$), as obtained from rhyolite-MELTS, can be directly transformed into an activity ($a_{{{\text{TiO}}_{2} }}$) value using the equation:

3 $$a_{{{\text{TiO}}_{2} }} = {\text{e}}^{{\left( {\frac{{ - A_{{{\text{TiO}}_{2} }} }}{R\cdot\;T}} \right)}} ,$$

where *R* is the gas constant (J mol^−1^ K^−1^) and *T* is the temperature (K).

The same holds true for SiO_2_. In case of Perple_X, the activity value can be calculated from chemical potential ($\mu_{{{\text{TiO}}_{2} }}$) and the Gibbs free enthalpy of rutile ($G_{{{\text{TiO}}_{2} }}$) values using the equation:

4 $$a_{{{\text{TiO}}_{2} }} = {\text{e}}^{{ - \left( {\frac{{G_{{{\text{TiO}}_{2} }}^{P,T} - \mu_{{{\text{TiO}}_{2} }}^{P,T} }}{R \cdot T}} \right)}} .$$

For Perple_X, the following mixing models were used: orthopyroxene, clinopyroxene, biotite, and melt (Powell and Holland 1999); feldspar (Benisek et al. 2010); ilmenite; and magnetite (Andersen and Lindsley 1988). Perple_X has the disadvantage that it does not consider the solubility of Ti in a granitic melt.

### Zircon solubility thermometry

Three models are available (Watson and Harrison 1983; Boehnke et al. 2013, Gervasoni et al. 2016); however, studies have shown they yield slightly different results (see Gervasoni et al. 2016 for comparison of the models). For this study, we preferred the model of Watson and Harrison (1983), because the temperature results appear to be more consistent with granitic melting experiments. For a haplogranitic melt near the ternary minimum (680 °C at 200 MPa water pressure; Johannes and Holtz 1991), the Zr solubility would be ~ 40 ppm according to Watson and Harrison (1983), ~ 83 ppm according to Boehnke et al. (2013) and ~ 95 ppm according to Gervasoni et al. (2016). Very-low-T granites with near minimum melt compositions (including fractionated and unfractionated subtypes—see Finger and Schiller 2012) commonly have Zr contents around 40 ppm and even lower (e.g., Williamson et al. 1996; Nabelek et al. 1992). This matches best with the Watson and Harrison’s (1983) model.

According to Watson and Harrison (1983), the relationship between *T* and the amount of dissolved Zr in a Zr-saturated silicate melt is:

5 $$\ln D^{\text{Zircon/Melt}} = - 3.8 - \left[ {0.85\left( {M - 1} \right)} \right] + \frac{12900}{T},$$

with *M* being a composition parameter given by the (molecular) ratio:

6 $$M = \frac{{x_{\text{Na}} + x_{\text{K}} + 2x_{\text{Ca}} }}{{x_{\text{Si}} \cdot\;x_{\text{Al}} }}.$$

Using Eq. (5) in combination with crystallization modeling by rhyolite-MELTS allows ideal magmatic zircon crystallization curves (*T* vs. zircon crystallization rate) to be calculated from whole-rock geochemistry data (Harrison et al. 2007; Ickert et al. 2011; Burke 2017). The calculation procedure is not trivial, because the decreasing melt proportion during granite crystallization has to be taken into account. In addition, *M* values do not remain constant. The approach recommended here is to initially model the major mineral crystallization history of the granite under study in steps of 10 °C by means of rhyolite-MELTS, monitoring both the melt proportion and its melt composition. The amount of dissolved Zr in the melt can then be calculated for every temperature step using the Watson and Harrison’s (1983) model, and these data points can finally be fitted by a logarithmic function. In combination with the Zr whole-rock data, *T* at which the first (autocrystic) zircon will ideally form in a given granite [*T*_Zr(*M*)_], and *T* at which 10, 20, or 50% of the zirconium will have crystallized as zircon (assuming that zircon is the only Zr carrier mineral and that all Zr was initially dissolved in the melt phase) can be obtained. Note that *T*_Zr(*M*)_ can be higher than the *T*_Zr_ saturation value of Watson and Harrison (1983) if a magma contains larger amounts of a restitic (or peritectic) major mineral component. Ideally, the theoretical zircon crystallization curve of a granite (*T* vs. percent of crystallized zircons) should be identical to the measured range of Ti-in-zircon temperatures (Ickert et al. 2011).

In conjunction with $a_{{{\text{TiO}}_{2} }}$– and $a_{{{\text{SiO}}_{2} }}$–*T* functions derived from rhyolite-MELTS or Perple_X, a detailed, *T*-dependent, $a_{{{\text{TiO}}_{2} }}$ and $a_{{{\text{SiO}}_{2} }}$ correction of Ti-in-zircon temperatures can be made that is particularly useful for granites, where $a_{{{\text{TiO}}_{2} }}$ and $a_{{{\text{SiO}}_{2} }}$ potentially changed during zircon crystallization.

To begin, we use a set of Ti contents measured in zircons from the Strážný granite from the South Bohemian Batholith to highlight the $a_{{{\text{TiO}}_{2} }}$ and $a_{{{\text{SiO}}_{2} }}$ issue and to introduce our methodological approach. Subsequently, we undertake a broader analysis of the variation of $a_{{{\text{SiO}}_{2} }}$ and $a_{{{\text{TiO}}_{2} }}$ in granitic systems, as a function of magma composition, *P* and *T*, based on different published granite compositions.

## The Strážný granite

### Zircon analysis results and uncorrected Ti-in-zircon temperatures

The set of LA–ICP–MS zircon analyses, which are presented here, was obtained on a handpicked, slightly ground and polished zircon grain mount from a granite sample from the Strážný Massif in the Czech part of the South Bohemian Batholith (Klomínský et al. 2010; Žák et al. 2014). The investigated granite belongs to the weakly peraluminous, I/S-transitional Weinsberg granite suite of the South Bohemian Batholith (Frasl and Finger 1991), which formed by fluid-absent melting of biotite–quartz-plagioclase assemblages in the lower crust, at temperatures between 850 and 900 °C (Finger and Clemens 1995). The Weinsberg granite of the Strážný Massif (termed here the Strážný granite) is a coarse-grained biotite granite with porphyritic K-feldspars of up to 5 cm length. Accessory minerals include ilmenite, apatite, monazite, and zircon. The SiO_2_ content of the Strážný granite varies between 65 and 73 wt%. The granite is slightly peraluminous, alkali–calcic, and ferroan (Frost et al. 2001).

The zircon analyses were carried out at Boise University, using a Thermo-Electron X-Series II quadrupole ICPMS and a New Wave Research UP-213 Nd:YAG UV (213 nm) laser ablation system. Fifty-four zircon grains were targeted with a spot size of ~ 25 µm. The analysis protocol followed Rivera et al. (2013) and was specifically designed to capture trace-element concentrations, Ti-in-zircon concentrations for thermometry, and U–Th–Pb isotope ratios within the same spot analysis. Dwell times were 5 ms for Si and Zr; 100 ms for ^49^Ti and ^207^Pb, 40 ms for ^238^U, ^232^Th, ^202^Hg, ^204^Pb, ^206^Pb, and ^208^Pb isotopes and 10 ms for all other HFSE and REE elements, for a total sweep time of 750 ms. A 60 s analysis (15 s gas blank, 45 s ablation with 5 Hz laser pulse and 14 J cm^−2^ fluence) excavated a pit approximately 25 µm deep. Background count rates for each analyte were obtained prior to each spot analysis and subtracted from the raw count rates. For concentration calculations, average background-subtracted count rates for each analyte were internally normalized to ^29^Si, and calibrated with respect to the primary standards NIST SRM-610 and SRM-612 glasses. Secondary standards included USGS BIR-1 and BCR-2 glasses and the AUS-Z2 zircon megacryst (Kennedy 2011). The analytical point error is better than 1 ppm for Ti which is equivalent to ± 10 °C.

Table 1 shows the range of Ti contents that were measured in the zircon crystals. They vary from 2.5 to 17.3 ppm and correspond to uncorrected Ti-in-zircon temperatures of 629–800 °C (excluded from Table 1 are 11 analyses of non-autocrystic zircon cores with old U–Pb ages). A geochemical analysis of the granite sample KV829 is given in Table 2. The whole-rock Zr content is 309 ppm and corresponds to a zircon saturation temperature (*T*_Zr_) of 841 °C (Watson and Harrison 1983). Overall, a negative SiO_2_–Zr covariation is seen in the whole-rock data for the Strážný granite. Thus, complete ZrSiO_4_ saturation can be assumed during magmatic crystallization (Chappell et al. 1998; Kemp et al. 2005), which is consistent with the preservation of inherited zircon. *T*_Zr_ should, thus, give an estimate of the peak magma temperature. However, the presence of older zircons should also be taken into account in the peak temperature estimation. From the measured U–Pb ages, we estimated that there is < 10% older zircon, which is equivalent to a reduction of the Zr-saturation temperature of ≤ 7 °C, which is considered insignificant.

**Table 1:** Ti contents measured in zircons from sample KV-829 and Ti-in-zircon temperatures calculated according to Ferry and Watson (2007)

Grain	Ti (ppm)	*T* (°C) uncorr.	*T* (°C) corrected	Correction parameters/temperature effect			
				$a_{{{\text{SiO}}_{2} }}$	(°C)	$a_{{{\text{TiO}}_{2} }}$	(°C)
1	17.3	800	880	0.84 (− 18)		0.41 (+ 101)	
2	13.4	774	853	0.87 (− 13)		0.42 (+ 94)	
3	12.3	766	845	0.89 (− 12)		0.42 (+ 92)	
4	12.0	763	842	0.89 (− 11)		0.42 (+ 92)	
5	11.8	761	840	0.89 (− 11)		0.42 (+ 91)	
6	11.6	760	838	0.89 (− 11)		0.42 (+ 91)	
7	11.1	756	834	0.90 (− 10)		0.42 (+ 90)	
8	10.3	748	826	0.91 (− 9)		0.42 (+ 88)	
9	10.2	748	826	0.91 (− 9)		0.42 (+ 88)	
10	9.9	745	823	0.92 (− 8)		0.42 (+ 87)	
11	9.9	745	823	0.92 (− 8)		0.42 (+ 87)	
12	9.7	743	820	0.92 (− 8)		0.42 (+ 87)	
13	9.6	742	819	0.92 (− 8)		0.42 (+ 87)	
14	9.4	741	818	0.92 (− 8)		0.43 (+ 86)	
15	9.4	740	818	0.92 (− 7)		0.43 (+ 86)	
16	9.4	740	818	0.92 (− 7)		0.43 (+ 86)	
17	9.4	740	817	0.92 (− 7)		0.43 (+ 86)	
18	9.3	739	816	0.92 (− 7)		0.43 (+ 86)	
19	9.3	739	816	0.92 (− 7)		0.43 (+ 86)	
20	8.7	733	810	0.93 (− 6)		0.43 (+ 84)	
21	8.6	732	808	0.93 (− 6)		0.43 (+ 84)	
22	8.6	732	808	0.93 (− 6)		0.43 (+ 84)	
23	8.5	731	808	0.93 (− 6)		0.43 (+ 84)	
24	8.5	731	808	0.94 (− 6)		0.43 (+ 84)	
25	8.5	731	807	0.94 (− 6)		0.43 (+ 84)	
26	8.4	730	807	0.94 (− 6)		0.43 (+ 84)	
27	8.4	730	807	0.94 (− 6)		0.43 (+ 84)	
28	8.2	728	804	0.94 (− 6)		0.43 (+ 83)	
29	8.0	725	802	0.94 (− 5)		0.43 (+ 83)	
30	7.6	721	797	0.95 (− 5)		0.43 (+ 82)	
31	7.6	721	797	0.95 (− 5)		0.43 (+ 82)	
32	7.0	713	789	0.96 (− 4)		0.43 (+ 80)	
33	6.8	711	787	0.96 (− 3)		0.43 (+ 80)	
34	6.6	709	784	0.97 (− 3)		0.43 (+ 79)	
35	6.4	706	782	0.97 (− 3)		0.43 (+ 78)	
36	6.3	704	779	0.97 (− 2)		0.43 (+ 78)	
37	6.0	701	776	0.98 (− 2)		0.43 (+ 77)	
38	5.4	691	765	0.99 (− 1)		0.44 (+ 75)	
39	5.3	690	764	0.99 (− 1)		0.44 (+ 75)	
40	5.3	689	763	0.99 (− 1)		0.44 (+ 75)	
41	4.1	668	739	1.00 (0)		0.44 (+ 71)	
42	4.0	666	736	1.00 (0)		0.44 (+ 70)	
43	2.5	629	692	1.00 (0)		0.45 (+ 63)

**Table 2:** Results of $a_{{{\text{TiO}}_{2} }}$ and $a_{{{\text{SiO}}_{2} }}$ calculations with rhyolite-MELTS and Perple_X using a variety of granite compositions

	1	2	3	4	5	6	7	8	9	10	11	12	13	14
	S-type	S-type	S-type	S-type	I-type	I-type	I-type	I-type	I-type	I-type	I-type	A-type	A-type	A-type
Petrography	Bt-G	Bt-Ms-G	Bt-Crd-G	Bt-Ms-G	Bt-G	Bt-GD	Bt-Amp-T	Bt-GD	Amp-Bt-GD	Bt-Amp-G	Bt-G	Bt-Amp-G	Amp-G	Amp-Ol-G
Chemical characterization	m,ac	m,ac	m,ac	m,a	m,ac	m,ca	m,ac	m,ac	m,ca	m,ca	m,ac	f,ca	f,ac	f,a
Orig. sample number	KV-829	Fi3–85	Fi16–85	Fi4–85	Fi8–85	Fi9–85	Fi45–86	UV-429	Z11	VB198	21	AB239	6E	AP8
SiO_2_	68.11	70.66	68.39	71.47	67.98	65.16	58.45	64.03	64.25	66.39	70.29	70.14	74.88	66.84
TiO_2_	0.65	0.34	0.50	0.23	0.44	0.50	1.41	0.46	0.57	0.50	0.24	0.49	0.07	0.38
Al_2_O_3_	13.84	14.51	14.61	14.32	14.66	16.61	16.19	16.45	15.09	13.45	14.59	13.02	11.71	14.13
Fe_2_O_3_	0.41	0.32	0.23	0.08	0.23	0.68	0.29	0.11	1.69	0.77	0.82	1.59	0.44	1.71
FeO	3.21	1.45	2.56	0.89	2.47	2.46	6.17	3.24	2.99	2.72	0.79	2.25	0.90	2.50
MnO	0.06	0.01	0.05	0.01	0.02	0.05	0.11	0.06	0.06	0.05	0.04	0.05	0.03	0.09
MgO	0.99	0.48	1.00	0.30	1.03	1.56	3.16	2.07	1.99	2.06	0.37	0.45	0.02	0.05
CaO	2.13	0.80	1.37	0.56	1.87	3.70	4.44	3.36	4.16	3.52	1.83	1.44	0.26	1.21
Na_2_O	2.94	3.01	2.99	3.40	3.55	3.76	2.96	2.33	4.07	3.02	3.90	3.44	4.09	4.55
K_2_O	4.67	5.41	5.31	5.75	4.74	2.51	3.84	4.86	2.12	4.52	4.11	4.11	4.62	5.54
H_2_O	3.00	3.00	3.00	3.00	3.00	3.00	3.00	3.00	3.00	3.00	3.00	3.00	3.00	3.00
Zr (ppm)	309	143	192	81	163	165	230	177	183	240	138	453	207	498
*T*_Zr_ (°C)	841	791	806	737	782	784	789	790	771	779	770	883	800	863
*T*_Zr(*M*)_ (°C)	850	807	824	773	810	835	855	834	829	833	798	883	818	875
*T*_Zr/2(M)_ (°C)	800	766	779	750	765	787	810	788	781	785	759	830	778	827
r-MELTS modelling														
Melt content at *T*_Zr(*M*)_ (%)	88	86	85	71	77	65	62	60	61	79	79	99	89	74
Major phases	Opx,Pl	Pl,Kfs,Opx	Pl,Opx	Pl,Kf,Qz,Opx	Pl,Opx	Pl,Opx	Pl,Opx,Cpx	Pl,Opx	Pl,Opx,Cpx	Pl,Opx,Cpx	Pl,Opx	–	Pl,Qz	Pl,Kfs
Accessory Ti-phases	Ilm,Mag	Ilm,Mag	Ilm	Ilm	Ilm	Ilm,Mag	Ilm	Ilm,Mag	Ilm,Mag	Ilm,Mag	Mag	Mag	Mag	Mag
$a_{{{\text{TiO}}_{2} }}$at *T*_Zr(*M*)_|*T*_Zr*/*2(*M*)_, 2 kbar	0.42|0.43	0.46|0.46	0.47|0.49	0.49|0.47	0.45|0.47	0.49|0.51	0.38|0.39	0.51|0.49	0.45|0.41	0.47|0.48	0.37|0.47	0.28|0.36	0.11|0.15	0.20|0.21
$a_{{{\text{TiO}}_{2} }}$ at *T*_Zr(*M*)_|*T*_Zr/2(*M*)_, 5 kbar	0.47|0.47	0.52|0.52	0.47|0.56	0.55|0.51	0.53|0.53	0.54|0.52	0.38|0.38	0.56|0.57	0.50|0.5	0.50|0.54	0.45|0.53	0.32|0.43	0.15|0.19	0.22|0.23
Perple_X modelling														
Melt content at *T*_Zr(*M*)_ (%)	86	90	94	79	88	68	61	74	62	79	88	91	94	88
Major phases	Opx,Pl	Bt,Crd,Pl	Bt,Pl	Kf,Bt,Crd	Bt,Pl	Bt,Pl	Opx,Pl,Bt,Amp	Bt,Pl, Crd	Pl,Opx, Cpx	Cpx,Opx, Pl	Opx,Pl	Pl	Cpx	Cpx
Accessory Ti-phases	Ilm,Mag	Ilm,Mag	Ilm,Mag	Ilm,Mag	Ilm,Mag	Ilm,Mag	Ilm	Ilm,Mag	Ilm,Mag	Ilm,Mag	Ilm,Mag	Ilm,Mag	Mag	Ilm,Mag,Ttn
$a_{{{\text{TiO}}_{2} }}$ at *T*_Zr(*M*)_*|T*_Zr/2(*M*)_, 2 kbar	0.45|0.45	0.49|0.47	0.48|0.49	0.50|0.49	0.47|0.47	0.50|0.53	0.45|0.47	0.50|0.57	0.51|0.56	0.51|0.54	0.53|0.59	0.51|0.50	0.56|0.53	0.57|0.54
$a_{{{\text{TiO}}_{2} }}$ at *T*_Zr(*M*)_|*T*_Zr*/*2(*M*)_, 5 kbar	0.48|0.48	0.51|0.51	0.57|0.51	0.54|0.54	0.52|0.49	0.58|0.55	0.53|0.52	0.52|0.59	0.52|0.55	0.59|0.55	0.56|0.62	0.49|0.49	0.40|0.54	0.57|0.52

Input data refer to published granite analyses, but include normalization to 100% after assuming an H_2_O content of 3%

*G* granite, *GD* granodiorite, *T* tonalite; mineral abbrevations after Whitney and Evans (2010), *f* ferroan, *m* magnesian, *ca* calc-alkalic, *ac* alkali-calcic, *a* alkalic (Frost et al. 2001), *T*_*Zr*_ zircon saturation *T*., *T*_*Zr(M*)_ zircon saturation *T*. corrected with melts, *T*_*Zr/2M*_ *T*_Zr(*M*)_at 50% whole rock Zr;

According to rhyolite-MELTS, sample KV829 was not fully molten at *T*_Zr_ with an assumed bulk content of 3% H_2_O. The minerals at this stage are given as oligoclase, ortho-pyroxene, ilmenite, and magnetite. As these phases do not incorporate appreciable amounts of Zr, and taking into account the reduced amount of melt (87%), *T*_Zr_ must be slightly upward corrected from 841 to 850 °C (*T*_Zr(*M*)_). Independent of these minor uncertainties, we note that the zircon saturation model gives a much higher temperature than the uncorrected Ti-in-zircon thermometer (Table 1). Even the highest obtained Ti-in-zircon *T* value of 800 °C (analysis 1 in Table 1) remains ~ 50 °C below *T*_Zr(*M*)_.

### Calculation of $a_{{{\text{TiO}}_{2} }}$ and $a_{{{\text{SiO}}_{2} }}$

Like most granites, the studied sample KV829 does not contain rutile. The major Ti-carrier minerals identified in thin section are ilmenite and biotite. The rhyolite-MELTS software indicates the following crystallization sequence for the rock at 2 kbar and a water content of 3 wt%: 7% plagioclase, 5% orthopyroxene, 0.5% magnetite, 0.5% ilmenite at 850 °C, K-feldspar-in at 783 °C, and quartz-in at 760 °C. As stated in Gualda et al. (2012), biotite is not dependably modelled by rhyolite-MELTS. In our calculation, biotite does not occur above the solidus, although magmatic biotite is obviously present in the sample. We will discuss these issue in Sect. 5.1.

**Fig. 1:**
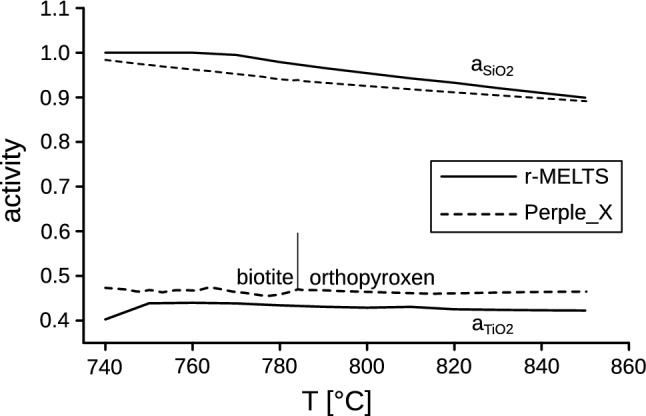
$a_{{{\text{TiO}}_{2} }}$ and $a_{{{\text{SiO}}_{2} }}$ vs. *T* functions for granite KV829 as calculated with rhyolite-MELTS and Perple_X

Transforming the TiO_2_ affinity values, as obtained from rhyolite-MELTS, into activity values (Eq. 3), we get $a_{{{\text{TiO}}_{2} }}$ = 0.42 at 850 °C and $a_{{{\text{TiO}}_{2} }}$ = 0.44 at 750 °C. The $a_{{{\text{TiO}}_{2} }}$–*T* function is shown in Fig. 1. An attempt to model $a_{{{\text{TiO}}_{2} }}$ independently with Perple_X leads to a very similar result (Fig. 1). The relatively low TiO_2_ activity values of 0.4–0.5, which are indicated by both software programs, would necessitate a significant upward correction of Ti-in-zircon temperatures, in the order of 80 °C. The Ti-in-zircon temperatures would then be shifted into a range that is very consistent with the zircon saturation temperature (Table 1).

Quartz crystallizes late in the paragenesis of sample KV829 (quartz-in at ~ 760 °C). Therefore, the $a_{{{\text{SiO}}_{2} }}$ values will remain below unity over a large part of the magmatic zircon crystallization range. Figure 1 shows the variation of $a_{{{\text{SiO}}_{2} }}$ as a function of *T*. According to rhyolite-MELTS, $a_{{{\text{SiO}}_{2} }}$ steadily increases with falling temperature from ~ 0.88 at 850 °C to ~ 0.95 at 800 °C before reaching unity at 760 °C (quartz-in). The Perple_X calculation gives a similar result (Fig. 1). The $a_{{{\text{SiO}}_{2} }}$ correction effect on the Ti-in-zircon temperatures can be taken from Table 1. The highest *T* zircons require the largest correction of 10–20 °C, but this is significantly less than the $a_{{{\text{TiO}}_{2} }}$-correction (Table 1).

### A useful test: comparing the Ti-in-zircon temperature range of a granite with its ideal magmatic zircon crystallization temperature distribution curve

**Fig. 2:**
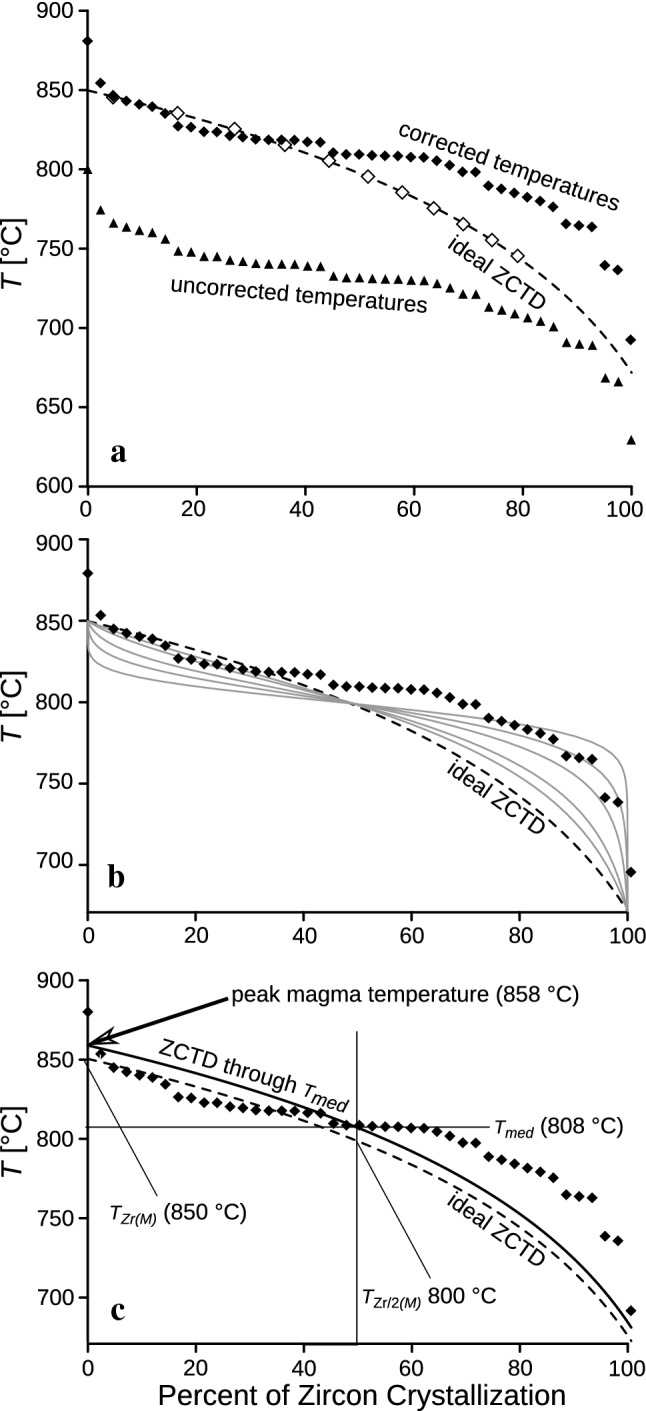
**a** Ideal “zircon crystallization temperature distribution” (ZCTD) for sample KV829 in comparison with the measured Ti-in-zircon temperatures (uncorrected vs. corrected values, using a *T*-dependent $a_{{{\text{TiO}}_{2} }}$ and $a_{{{\text{SiO}}_{2} }}$ correction routine—see Table 1). **b** Measured Ti-in-zircon temperatures for sample KV829 ($a_{{{\text{TiO}}_{2} }}$ and $a_{{{\text{SiO}}_{2} }}$ corrected) in comparison with the ideal ZCTD and model curves (grey) that simulate an overrepresentation of average zircons (see text). **c** Measured Ti-in-zircon temperatures for sample KV829 and calculated *T*_Zr(*M*)_–*T*_Zr*/2(M)*_ relations. A robust estimation of *T*_max_ (theoretical peak magma temperature in the case of zircon saturation) is possible by constructing a parallel curve to the ideal ZCTD through *T*_med_, the intersection of which with the ordinate gives *T*_max_

Figure 2a shows the “zircon crystallization temperature distribution” (ZCTD) (Ickert et al. 2011) for sample KV829 as modelled on the basis of geochemistry, in synopsis with the corrected and uncorrected Ti-in-zircon temperatures (arranged by decreasing temperature). It can be seen that the $a_{{{\text{TiO}}_{2} }}$ and $a_{{{\text{SiO}}_{2} }}$ corrections have shifted the Ti-in-zircon temperatures into a range that is consistent with the zircon crystallization model of Watson and Harrison (1983).

The peak temperature of a granitic magma is commonly of greatest interest for magmatic petrology. However, its direct determination using Ti-in-zircon thermometry is difficult. The highest measured Ti content in magmatic (autocrystic) zircon should give this peak temperature (Siégel et al. 2018). However, it is inherently difficult to accurately measure the maximum and minimum Ti content of a magmatic zircon population by current, commonly used zircon analysis techniques due to insufficient spatial resolution (Ickert et al. 2011). Ti concentrations in zircon are normally measured using an ion probe or a Laser–ICP–MS with 20–30 µm measuring spot sizes. Although accessory zircons in granites are commonly 50–200 µm in size, they are generally internally finely zoned with early magmatic high temperature cores and late-magmatic low-temperature rims (Ickert et al. 2011). Measuring these fine zones with 20–30 µm spot sizes is fraught with difficulty. Thus, measured Ti amounts often contain some averaging effect; intermediate compositions and temperature data will be overrepresented.

The sigmoidal shape of the Ti-in-zircon temperature curve in Fig. 2a likely reflects a method-inherent sampling bias towards medium-T zircons. We have tried to replicate such a sampling bias effect with a model calculation. The ideal ZCTD for sample KV829 was used as starting situation and then adjusted using Gaussian probability models with different standard deviations. It can be seen that the original logarithmic curve is distorted to a sigmoidal shape in these models similar to the observed distribution (Fig. 2b).

**Fig. 3:**
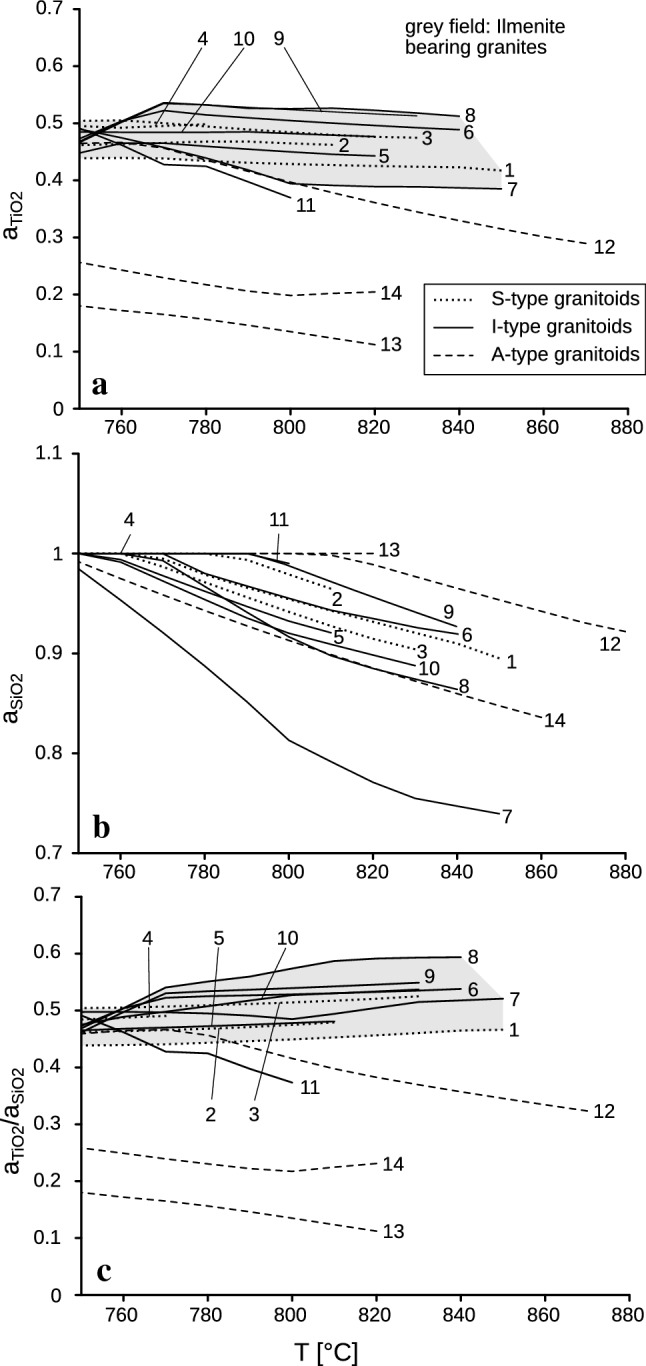
$a_{{{\text{SiO}}_{2} }}$–*T* and $a_{{{\text{TiO}}_{2} }}$–*T* functions as calculated for different granite compositions with rhyolite-MELTS (numbers refer to granite analyses given in Table 2)

Thus, instead of merely relying on the measured maximum Ti, users of the Ti-in-Zr thermometer should also try to estimate the peak magma temperature from the median Ti content of a zircon population, which is a more robust parameter. We propose here that the median Ti-in-zircon temperature (*T*_med_, i.e., the middle value of the temperature range) should be routinely reported in granite studies. Theoretically, exactly half (by mass) of a magmatic zircon population should have crystallized at *T*_med_, and half of the zirconium is still dissolved in the melt fraction. The construction of an ideal “zircon crystallization temperature distribution” from whole-rock chemistry data (Fig. 3c) provides the means to estimate the maximum temperature of a granite melt from the *T*_med_ value. Due to the logarithmic nature of the ZCTD, the difference between *T*_med_ and *T*_max_ is constant for a given sample, independent of absolute Zr-content or melt temperature, see Sect. 2 for the calculation procedure.

A comparison of measured Ti-in-zircon temperatures with the ZCTD curve of a sample can greatly help to recognize anomalous measurements from, for instance, antecrystic or xenocrystic zircons (Siégel et al. 2018; Claiborne et al. 2010) or simply bring to light inaccurate or contaminated analyses: For example, with respect to our case study (sample KV829, Table 1, Fig. 2), we observe that the highest measured Ti value (17.3 ppm, 880 °C after correction) is an outlier of unclear significance. It is possible that the laser beam hit a small Ti bearing inclusion, which was overlooked during data reduction. The second-highest Ti value (13.4 ppm; analysis 2 in Table 1) gives a temperature of 853 °C and lies very close to the theoretical zircon crystallization curve.

## Modeling $a_{{{\text{TiO}}_{2} }}$–*T* and $a_{{{\text{SiO}}_{2} }}$–*T* functions for different granite types

### Procedure and assumptions

Granitic magmas (sensu lato) show considerable compositional variations in terms of their silica and alkali-element contents (Middlemost 1994), their peraluminousity and maficity (Chappell and White 1974, Clemens and Stevens 2012), and their Fe/Mg and (Na+K)/Ca ratios (Frost et al. 2001). Our intention was to investigate, by means of rhyolite-MELTS and Perple_X, to what extent $a_{{{\text{TiO}}_{2} }}$ and $a_{{{\text{SiO}}_{2} }}$ values can vary in granitic systems as a function of magma composition. To this end, we have utilized the following selection of published granite data (numbers refer to Table 2; the designation of the rocks to the S-, I-, or A-type granite group is based on the original papers, which are cited below):

- 1–4: variably SiO_2_-rich, moderately to strongly peraluminous S-type granites from the Variscan South Bohemian Batholith (Liew et al. 1989; Fuchs and Thiele 1968).
- 5–8: various, medium-K to high-K, I-type granitoids (incl. granite, granodiorite, and tonalite) from the South Bohemian Batholith (Liew et al. 1989; Fuchs and Thiele 1968) and the Austrian Hohe Tauern Batholith (Finger and Steyrer 1988).
- 9: a volcanic-arc granodiorite (I-type) from the Zagros orogen in Iran (Alaminia et al. 2013).
- 10: fractionated biotite-amphibole granite from the Boggy Plain high-T, I-type suite (Wyborn et al. 1987; Chappell et al. 1998).
- 11: a magnetite-series, I-type granodiorite from the Tuolumne intrusive complex (Bateman and Chappell 1979).
- 12–14: three representatives of A-type granites from the Lachlan Fold belt (King et al. 1997), the Evisa complex in Corsica (Whalen et al. 1987), and the White Mountain Complex (Eby et al. 1992).

Zircon crystallization curves were constructed for all 14 granites following the procedure described in Sect. 2. The *T*_Zr/2_ value in Table 2 gives the temperature, at which half of the whole-rock Zr content is still dissolved in the melt phase, i.e., after 50% of the zircons precipitated. For the sake of simplicity complete zircon saturation was assumed for all rocks, and any possible occurrence of non-autocrystic zircon was ignored, as these factors have no influence on the $a_{{{\text{SiO}}_{2} }}$ and $a_{{{\text{TiO}}_{2} }}$ estimates. Interestingly, the modeling suggests that appreciable amounts (1–39%) of solid phases were present in nearly all of the granitoids at the estimated peak temperatures [*T*_Zr(*M*)_ values in Table 2]. We would interpret this in terms of restite or peritectic phase contents (Chappell et al. 1987; Stevens et al. 2007; Clemens and Stevens 2012) or due to crystal accumulations.

### $a_{{{\text{TiO}}_{2} }}$ values

Using the rhyolite-MELTS software program, 10 of the 14 investigated granites are modelled to have crystallized in a surprisingly narrow $a_{{{\text{TiO}}_{2} }}$ range of 0.5 ± 0.1. All these granites with $a_{{{\text{TiO}}_{2} }}$ ~ 0.5 (1–11 in Table 2) have in common that they contain ilmenite in the rhyolite-MELTS calculation. The $a_{{{\text{TiO}}_{2} }}$–*T* functions of these ilmenite-bearing granites are always flat (Fig. 3a), and the $a_{{{\text{TiO}}_{2} }}$ values for 5 kbar are only insignificantly higher than for 2 kbar (Table 2).

The three A-type granite examples (12–14 in Table 2) and the I-type granite number 11 (a relatively strongly oxidized granite with Fe_2_O_3_ > FeO) give clearly lower $a_{{{\text{TiO}}_{2} }}$ values of between 0.15 and 0.45 and are ilmenite free in the rhyolite-MELTS calculation at *T*_zr(*M*)_. Notably, the $a_{{{\text{TiO}}_{2} }}$–*T* functions of these four samples show a pronounced negative slope (Fig. 3a). The $a_{{{\text{TiO}}_{2} }}$ values for 5 kbar are again slightly higher than for 2 kbar (Table 2).

The calculation with Perple_X gives $a_{{{\text{TiO}}_{2} }}$ values of 0.5–0.6 for granites 1–11 (Table 2). These values are only slightly higher than the rhyolite-MELTS-based $a_{{{\text{TiO}}_{2} }}$ values. However, importantly, Perple_X does not reproduce the low $a_{{{\text{TiO}}_{2} }}$ values that were calculated with rhyolite-MELTS for granites 12–14 and gives much higher $a_{{{\text{TiO}}_{2} }}$ values for these three rocks (0.4–0.6 between 700 and 800 °C). Note also that Perple_X calculates ilmenite contents for two of these granites, unlike rhyolite-MELTS. It is possible that Perple_X systematically overestimates ilmenite stability, because the Ti content of the melt phase is left unconsidered.

### $a_{{{\text{SiO}}_{2} }}$ values

The rhyolite-MELTS software program indicates a considerable spread of the SiO_2_ activity for granites at the onset of magmatic zircon crystallization (Fig. 3b). At 2 kbar, values range from $a_{{{\text{SiO}}_{2} }}$= 1 in the most SiO_2_-rich granites to only 0.75 in the least felsic I-type tonalite (number 7 in Table 2). During ongoing magmatic crystallization, i.e., with falling *T*, $a_{{{\text{SiO}}_{2} }}$ approaches unity in all cases. The $a_{{{\text{SiO}}_{2} }}$–*T* curves at 5 kbar are parallel shifted to slightly higher activity values. The calculation of $a_{{{\text{SiO}}_{2} }}$ values with Perple_X gives nearly the same results (see e.g., Fig. 1).

### $a_{{{\text{TiO}}_{2} }}$*/*$a_{{{\text{SiO}}_{2} }}$ net correction effects for Ti-in-zircon thermometry

**Fig. 4:**
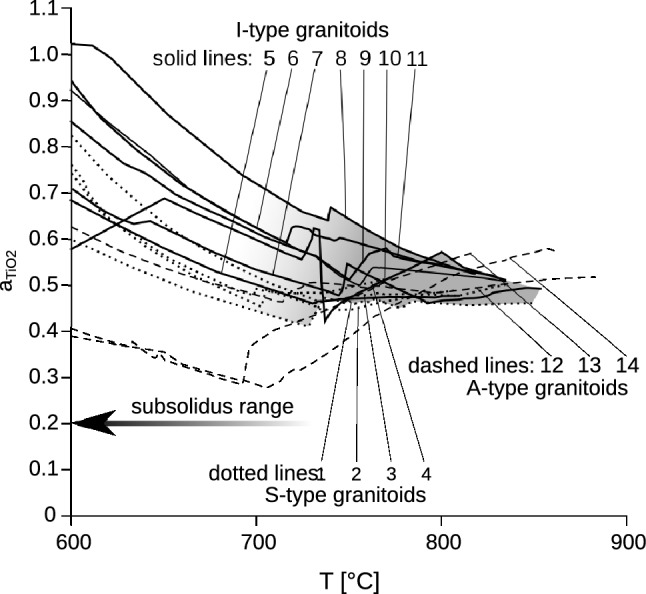
$a_{{{\text{TiO}}_{2} }}$–*T* functions for the granites from Table 2 calculated with Perple_X. *P* = 2 kbar. The following mixing models were used: orthopyroxene, clinopyroxene, biotite, and melt (Powell and Holland 1999); feldspar (Benisek et al. 2010); ilmenite; and magnetite (Andersen and Lindsley 1988)

As can be seen from Eq. (2), $a_{{{\text{TiO}}_{2} }}$ and $a_{{{\text{SiO}}_{2} }}$ exert an opposite correction effect on Ti-in-zircon temperatures and it has been speculated that both may widely cancel out in a number of cases (Ferry and Watson 2007). However, according to our calculations, the difference from unity is always much larger for $a_{{{\text{TiO}}_{2} }}$ than for $a_{{{\text{SiO}}_{2} }}$. Thus, a large net correction generally remains (Fig. 3c), although the $a_{{{\text{TiO}}_{2} }}$*/*$a_{{{\text{SiO}}_{2} }}$ net correction will be, in general, a little smaller than the $a_{{{\text{TiO}}_{2} }}$ correction alone. According to rhyolite-MELTS, ilmenite-bearing granitic rocks would require a more or less constant $a_{{{\text{TiO}}_{2} }}$*/*$a_{{{\text{SiO}}_{2} }}$ net correction for the Ti-in-zircon thermometer in the order of 0.5. This would correspond to a temperature correction of approximately + 70 °C. According to Perple_X, the $a_{{{\text{TiO}}_{2} }}$*/*$a_{{{\text{SiO}}_{2} }}$ net correction would be in the range of 0.5–0.6 for most granites, equivalent to a temperature correction of approximately + 60 °C (Table 1). The net corrections for 2 and 5 kbar remain approximately the same, because both $a_{{{\text{TiO}}_{2} }}$ and $a_{{{\text{SiO}}_{2} }}$ sympathetically increase from 2 to 5 kbar (Fig. 4).

A-type granites and a few special I-type granites may require significantly greater $a_{{{\text{TiO}}_{2} }}$*/*$a_{{{\text{SiO}}_{2} }}$ net corrections for Ti-in-zircon thermometry of between 0.1 and 0.5, which would correspond to temperature corrections in the order of + 100 to + 200 °C.

## Discussion

### How reliable are the $a_{{{\text{TiO}}_{2} }}$ estimates from rhyolite-MELTS and Perple_X?

Our calculations with rhyolite-MELTS and Perple_X indicate that zircon crystallization in granites commonly takes place at $a_{{{\text{SiO}}_{2} }}$ values of close to 1 and at $a_{{{\text{TiO}}_{2} }}$ values of around 0.5. This would suggest that Ti-in-zircon temperatures for granites have to be significantly upward corrected relative to the original TiO_2_- and SiO_2_-saturated calibration of the thermometer, in the order of + 70 °C. Looking through the literature, we observe that many applications of Ti-in-zircon thermometry to granites so far (including the most recent papers) involved no or only a minor $a_{{{\text{TiO}}_{2} }}$*/*$a_{{{\text{SiO}}_{2} }}$ correction and could thus bear a significant systematic error (e.g., Sepidbar et al. 2018; Roberts et al. 2018; Chen et al. 2018; Grajales-Nishimura et al. 2018; Langone et al. 2018; Steshenko et al. 2017; Zhang et al. 2018; Shabanian et al. 2017).

**Fig. 5:**
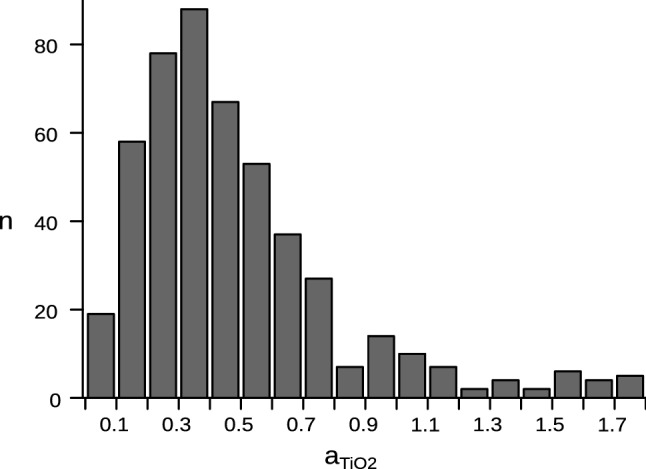
Histogram of $a_{{{\text{TiO}}_{2} }}$ values of experimentally produced granitoid melts. Values were recast from the TiO_2_ contents of these melts (data source: Gao et al. 2016) in comparison with the TiO_2_ solubility (Hayden and Watson 2007)

The crucial question is, of course, how accurate and reliable are the activity values from rhyolite-MELTS and Perple_X and can they be confirmed by other methods? For instance, Hayden and Watson (2007) have suggested a method for assessing the TiO_2_ activity of felsic volcanic rocks by comparing the TiO_2_ content of volcanic glass with that of a rutile-saturated melt (Ryerson and Watson 1987). An analogous approach can be made using the TiO_2_ contents of rapidly quenched melts from melting experiments on crustal rocks. Gao et al. (2016) present a comprehensive compilation of such data. An evaluation of this data shows that experimentally produced granitoid melts are commonly strongly TiO_2_-undersaturated in comparison with rutile-saturated melt systems (Hayden and Watson 2007). Most have TiO_2_ contents that correspond to $a_{{{\text{TiO}}_{2} }}$ values of around or just below 0.5 (Fig. 5), and this agrees very well with the rhyolite-MELTS calculations performed in the present paper for a series of granitic rocks.

Most granites do not represent true melt compositions and can contain restitic, peritectic or antecrystic Ti minerals (Clemens and Stevens 2012). Therefore, $a_{{{\text{TiO}}_{2} }}$ values are, in general, not deducible from TiO_2_ whole-rock concentrations. However, in the case of A-type granites 13 and 14 in Table 2, it is obvious from their low TiO_2_/Zr ratios that they must have been severely TiO_2_ undersaturated at near-liquidus conditions. Thus, the particularly low $a_{{{\text{TiO}}_{2} }}$ values of 0.1–0.3, as calculated by rhyolite-MELTS, are confirmed.

Gualda and Ghiorso (2013) have estimated the activity of TiO_2_ for a large number of rhyolites and dacites from different volcanic centres, based on mineral chemistry data of coexisting magnetite and ilmenite. Their results show that intermediate $a_{{{\text{TiO}}_{2} }}$ values, in the range of 0.5 ± 0.2, are very common in felsic magmatic systems. However, examples of volcanic rocks with systematically higher $a_{{{\text{TiO}}_{2} }}$ between 0.7 and 0.9 have also been reported. Gualda and Ghiorso (2013) state that such high-$a_{{{\text{TiO}}_{2} }}$ magmas are always highly oxidized with oxygen fugacity values well above the Ni–NiO oxygen buffer. For one of these high-$a_{{{\text{TiO}}_{2} }}$ rocks (the Fish Canyon Tuff—Bachmann et al. 2002), we calculated an $a_{{{\text{TiO}}_{2} }}$ value of ~ 0.7 for 750 °C and 0.9 for 650 °C using Perple_X, which is in good agreement with the Gualda and Ghiorso (2013) values. An attempt to calculate $a_{{{\text{TiO}}_{2} }}$ with rhyolite-MELTS failed, because the calculation did not converge at *T* < 750 °C. The example of the Fish Canyon Tuff shows that certain oxidized rhyolite magmas can crystallize at an $a_{{{\text{TiO}}_{2} }}$ of close to 1. We would expect that intrusive equivalents with similarly high $a_{{{\text{TiO}}_{2} }}$ exist as well. However, considering published Fe^3+^ contents, we tend to believe that such high $f_{{{\text{O}}_{ 2} }}$ and $a_{{{\text{TiO}}_{2} }}$ values are only rarely encountered among granites.

In their re-assessment of the Ti-in-zircon thermometer, Ferry and Watson (2007) have proposed that $a_{{{\text{TiO}}_{2} }}$ will commonly be between 0.6 and 0.9 in granitic systems. This $a_{{{\text{TiO}}_{2} }}$ estimate is a little higher than the values which we received from rhyolite-MELTS and Perple_X. Ferry and Watson (2007) quote two arguments in favor of the 0.6–0.9 $a_{{{\text{TiO}}_{2} }}$ range: first, they refer to the work of Ghent and Stout (1984), who calculated $a_{{{\text{TiO}}_{2} }}$ close to 1 for amphibolite facies (600 °C, 6 kbar) metapelitic rocks. However, we argue that the results of Ghent and Stout (1984) are difficult to extrapolate to granite crystallization conditions, because $a_{{{\text{TiO}}_{2} }}$ strongly decreases between 600 and 700 °C (Fig. 4). Therefore, it will be much lower at supra-solidus conditions than at 600 °C. The second argument of Ferry and Watson (2007) relies on the TiO_2_ measurements of Hayden and Watson (2007) in rhyolite glasses (as mentioned above). Most of these measurements would indicate $a_{{{\text{TiO}}_{2} }}$ values of 0.6–0.8, but there are also data that correspond to lower $a_{{{\text{TiO}}_{2} }}$ of 0.3–0.6. It could be that felsic volcanic systems have, on average, higher $a_{{{\text{TiO}}_{2} }}$ values than granites, due to a higher degree of fractionation and higher $f_{{{\text{O}}_{ 2} }}$ (Gualda and Ghiorso 2013).

With respect to the suitability of the rhyolite-MELTS model for the $a_{{{\text{TiO}}_{2} }}$ (and $a_{{{\text{SiO}}_{2} }}$) determination, one aspect may deserve additional discussion: rhyolite-MELTS models orthopyroxene and/or clinopyroxene as major mafic phases of granites and does not properly account for biotite and/or hornblende crystallization above the solidus, which is definitely a mismatch with nature. Using the Perple_X software, we have investigated whether biotite and hornblende crystallization modifies $a_{{{\text{TiO}}_{2} }}$ in a granitic magma relative to orthopyroxene and clinopyroxene parageneses. For instance, Perple_X suggests early ortho-pyroxene crystallization between 850 and 785 °C and biotite-in at 785 °C for our sample KV829 (Table 2). Only an insignificant change of the $a_{{{\text{TiO}}_{2} }}$ value is recorded at 785 °C in the $a_{{{\text{TiO}}_{2} }}$–*T* function (Fig. 1), implying that the presence or absence of biotite vs. orthopyroxene has little effect on the $a_{{{\text{TiO}}_{2} }}$. For sample VB198, Perple_X calculates clinopyroxene crystallization at 830 °C (in the absence of hornblende), whereas hornblende crystallization occurs upon further cooling. The $a_{{{\text{TiO}}_{2} }}$–*T* function (Fig. 4) shows no major change of $a_{{{\text{TiO}}_{2} }}$ at the hornblende-in stage. Therefore, based on these observations we would believe that the general underrating of biotite and hornblende in the rhyolite-MELTS model has no substantial influence on the $a_{{{\text{TiO}}_{2} }}$ calculation.

### An interesting test case: the Bishop Tuff

**Fig. 6:**
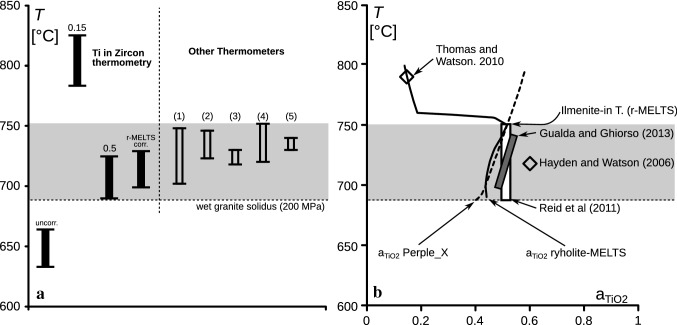
**a** Approximate liquidus–solidus temperature interval (grey) of the early Bishop Tuff magma as determined from various geothermometers: (1) Fe-Ti-oxide thermometer on crystal-poor samples of early Bishop Tuff (Hildreth and Wilson 2007). (2) δ^18^O Qz/Mt (eruptive sequence Ig1Ea; Bindeman and Valley 2002). (3) Ti in Qz (eruptive sequence Ig1Eb; Wark et al. 2007). (4) *T*_Zr_ of glass inclusions (early Bishop Tuff; Gualda and Ghiorso 2013). (5) *T*_Zr_ (early Bishop Tuff (Hildreth 1979; Watson and Harrison 1983). Shown for comparison are the results of Ti-in-zircon thermometry (eruptive sequence Ig1Eb; Chamberlain et al. 2013), using various corrections for Ti activity ($a_{{{\text{SiO}}_{2} }}$ is assumed to be 1). **b** Published TiO_2_-activity data for the Bishop Tuff in comparison to the $a_{{{\text{TiO}}_{2} }}$–*T* function calculated with rhyolite-MELTS (bold line) and Perple_X (dashed line)

This paper focuses on the application of Ti-in-zircon thermometry in granites, but a consideration of the well known Bishop Tuff in California is informative. The Bishop Tuff belongs to one of the best studied magmatic systems and a wealth of geochemical and mineral chemistry data is available on it, including Ti-in-zircon measurements. We refer, here, to a set of Ti-in-zircon data from the Early Bishop Tuff (eruptive sequence Ig1Eb) published in Chamberlain et al. (2013). This pumice-rich, phenocryst-poor ignimbrite is considered to represent a nearly pure rhyolite melt composition (Hildreth 1979). Various thermometers (Fe–Ti oxides; δ^18^O Qz/Mag; Ti in Qz; Zr solubility) constrain the liquidus–solidus temperature interval to lie between ~ 750 and 680 °C (Fig. 6).

A complication for Ti-in-zircon thermometry is the occurrence of sector-zoned zircons (Chamberlain et al. 2013). A non-stoichiometric incorporation of trace elements is observed in pyramidal zircon sectors (darker tips in CL images) indicating disequilibrium growth entrapment of surface impurities (Watson and Liang 1995; Watson 1996). The measured Ti-in-zircon temperatures range from 633 to 664 °C (uncorrected), excluding these sector zoned tips, which is far below the other estimates of the magmatic crystallization temperatures (Fig. 6). The necessity for an $a_{{{\text{TiO}}_{2} }}$ correction to these data has been pointed out by Chamberlain et al. (2013). However, they also emphasize that very different $a_{{{\text{TiO}}_{2} }}$ values are published for the rock (0.15–0.63), which would result in extremely different Ti-in-zircon temperatures (Fig. 6).

We calculate $a_{{{\text{TiO}}_{2} }}$ values of 0.47/0.48 at 730 °C, and 0.43/0.43 at 700 °C using rhyolite-MELTS and Perple_X, and the geochemical data of Hildreth (1979). The results confirm our hypothesis that ilmenite-bearing felsic magmas commonly have $a_{{{\text{TiO}}_{2} }}$ close to 0.5. Note that Gualda and Ghiorso (2013) and Reid et al. (2011) calculated similar $a_{{{\text{TiO}}_{2} }}$ values of 0.4–0.7 and 0.53 ± 0.1 for the Bishop Tuff from magnetite–ilmenite compositions. Hayden and Watson (2007) have estimated a similar $a_{{{\text{TiO}}_{2} }}$ value (0.6) based on TiO_2_ contents measured in volcanic glass. Thus, for the temperature range of 750–700 °C, all $a_{{{\text{TiO}}_{2} }}$ calculations are in good agreement. Figure 6 shows that the rhyolite-MELTS-based activity correction shifts the Ti-in-zircon temperatures into a range that is consistent with other published thermometric data. Interestingly, at *T *> 750 °C, rhyolite-MELTS calculations give significantly lower $a_{{{\text{TiO}}_{2} }}$ values, e.g., 0.15 at the calculated liquidus temperature of 780 °C (Thomas and Watson 2012). We observe a sharp change in the $a_{{{\text{TiO}}_{2} }}$–*T* behaviour at ~ 750 °C (Fig. 6), which coincides with the presence of ilmenite. The absence of ilmenite is calculated by rhyolite-MELTS at *T* > 750 °C. Calculations using Perple_X do not indicate this strong change in $a_{{{\text{TiO}}_{2} }}$. Ilmenite is stable at *T* > 750 °C, because the Ti content of the melt is not considered. Ilmenite forms phenocrysts in the Late Bishop Tuff. Therefore, $a_{{{\text{TiO}}_{2} }}$ values for a ilmenite saturated system must be used for correcting the Ti-in-zircon temperatures. This conclusion is also supported by the fact that a correction with $a_{{{\text{TiO}}_{2} }}$ = 0.15 (the value of Thomas and Watson 2012) would give unrealistically high Ti-in-zircon temperatures (Fig. 6). The example of the Bishop Tuff demonstrates that presence or absence of early ilmenite is an important criterion for making accurate $a_{{{\text{TiO}}_{2} }}$ determinations.

### Revisiting two other prominent Ti-in-zircon thermometric studies

In the light of our results, we revisit the work of Fu et al. (2008). They measured Ti contents in zircons from a series of igneous rocks and calculated Ti-in-zircon temperatures from these data. Fu et al. (2008) casted fundamental doubts on the validity of the Ti-in-zircon thermometer, arguing that the temperature estimates that they obtained for their samples were, in general, unrealistically low. We limit our comment here to only the data from granitic–tonalitic and rhyolitic–dacitic rocks. Indeed, they quote mean Ti-in-zircon temperatures (uncorrected) for these felsic to intermediate magmatic rocks that appear partly unrealistically low (e.g., 588 °C in one case). Moreover, single zircon analyses often provided temperature data below the wet granite solidus.

However, assuming that most rocks of granitoid composition crystallize at $a_{{{\text{TiO}}_{2} }}$ values of close to 0.5 (or even lower), the Ti-in-zircon temperatures of Fu et al. (2008) would have to be systematically upward corrected by at least 70 °C and, as such, would approach entirely plausible values. Peak temperature estimates for the rocks would then be between 680 and 900 °C (considering the $a_{{{\text{TiO}}_{2} }}$ correction and the fact that *T*_med_ values of Ti-in-zircon thermometry are generally 35–50 °C below peak magma temperatures). Thus, with regard to the granitoid rocks investigated in Fu et al. (2008), we see absolutely no reason to challenge the Ti-in-zircon thermometer per se. We would rather argue that the data in Fu et al. (2008) confirm the necessity of a significant $a_{{{\text{TiO}}_{2} }}$ correction when Ti-in-zircon thermometry is applied to granitoid rocks. We notice, though, that the Ti-in-zircon data of Fu et al. (2008) involve unusually large variations within single zircon populations, resulting in high standard deviations to the average Ti-in-zircon temperatures. Common *T*_max_–*T*_med_–*T*_min_ relationships of zircon crystallization should be considerably smaller (Fig. 2), so additional problems seem to be at play. More detailed reanalysis of some of the samples may allow further clarification.

Second, we want to comment briefly on the work of Harrison and Schmitt (2007), who conducted Ti-in-zircon thermometry on the renowned detrital Hadean (i.e., 4.4 Ga old) zircons from Jack Hills/Australia. Application of our standard correction of $a_{{{\text{TiO}}_{2} }}$*/*$a_{{{\text{SiO}}_{2} }}$ = 0.5 would shift their zircon formation temperatures from 678 ± 42 (mean Ti-in-zircon temperature and standard deviation according to Harrison and Schmitt 2007) to ~ 750 °C (Carley et al. 2014). Considering in addition the *T*_med_–*T*_max_ relationship, the parental magma could have had a peak temperature of closer to 800 °C, which would be absolutely compatible with fluid-absent muscovite and incipient biotite melting in the source rock (Clemens and Vielzeuf 1987). Peak magma temperatures could have been even above 800 °C if $a_{{{\text{TiO}}_{2} }}$ was lower than 0.5 or if the melt was Zr undersaturated. The contention of Harrison and Schmitt (2007) that these Hadean zircons likely precipitated from a water-saturated low-T silicate melt must thus be challenged.

## Conclusions

Without performing an $a_{{{\text{TiO}}_{2} }}$ correction, the application of the Ti-in-zircon thermometer to granitic rocks is presumably too imprecise for providing significant petrological information. To enhance the petrological significance of the thermometer and to reach a high comparability of data, we suggest here that the rhyolite-MELTS software (Gualda et al. 2012) should routinely be used for correcting Ti-in-zircon temperatures for granitoid rocks. It also allows quantifying the (comparably minor) influence of $a_{{{\text{SiO}}_{2} }}$. The method appears overall reliable and has the advantage that it is widely applicable, since geochemical data are commonly available (in cases where no Fe^3+^ data exist, various oxygen buffers can be used in calculation). Rhyolite-MELTS may be preferable to the Perple_X software, because it considers the Ti solubility in melts as well, but both software programs give, in general, comparable results (exceptions are Ti poor granites where the Ti is entirely in the melt at near liquidus conditions; for such situations, Perple_X erroneously calculates ilmenite and thus higher $a_{{{\text{TiO}}_{2} }}$ values).

A second suggestion for applied Ti-in-zircon thermometry would be to always quote the robust median Ti-in-zircon temperature (*T*_med_) of a magmatic zircon population and to model the peak magma temperature based on the ideal zircon crystallization temperature distribution. As a rule, *T*_med_ is about 35–50 °C below the peak magma temperature. Mismatches between the modelled peak magma temperature and the highest measured Ti-in-zircon temperatures must be taken as a hint that complications are at play (Siegel et al. 2018) and the data should be thoroughly reassessed.

We are of the optimistic view that, in the near future, Ti-in-zircon thermometry could achieve the capability to determine the magma temperature of granites with an uncertainty as low as approximately ± 20–30 °C, which is roughly twice the calibration uncertainty of the thermometer. To reach this, possible error sources and pitfalls (e.g., uncertainties in the $a_{{{\text{TiO}}_{2} }}$ estimation) must be carefully assessed through a number of case studies. These should involve a systematic comparison of Ti-in-zircon temperatures with independent estimations of peak magma temperatures based on Zr and REE solubility data or other suitable thermometers. However, for granitic systems with low $a_{{{\text{TiO}}_{2} }}$, the Ti-in-zircon thermometer could remain generally problematic because of the large influence of the $a_{{{\text{TiO}}_{2} }}$ correction.

A persisting uncertainty of the thermometer is the unquantified pressure effect, and experimental work on this field would be highly desirable to further refine the thermometer. The pressure effect may not be as great as feared by some authors (Ferry and Watson 2007), based on Δ*V* calculations of the ZrSiO_4_ and ZrTiO_4_ endmembers. Ti incorporation in zircon is most likely achieved by elastic lattice dilation without any change of the unit-cell volume (Blundy and Wood 1994). As the intrusion depths of most granites are similar, i.e., between 5 and 15 km (~ 2 to 5 kbar), a relative comparability of Ti-in-zircon temperatures will be given for most granite studies in any case.

When whole-rock data are missing, and for out-of-context zircons, a unified standard correction of + 70 °C reflecting a ratio of $a_{{{\text{TiO}}_{2} }}$_*/*_$a_{{{\text{SiO}}_{2} }}$ of ~ 0.5 may be a first order approximation for Ti-in-zircon thermometry. We believe that this + 70 °C correction gives good results for ilmenite-bearing granites, which are the most common granites worldwide. It seems better to rely on this simple approach rather than relying on uncorrected Ti-in-zircon temperature data. However, if geochemical data are available for a granite sample, one should always make the effort to determine $a_{{{\text{TiO}}_{2} }}$ and $a_{{{\text{SiO}}_{2} }}$ values using rhyolite-MELTS, the more so because the procedure is easy and rapid to perform.

## References

[CR1] Alaminia Z, Karimpour MH, Homam M, Finger F (2013). The magmatic record in the Arghash region (northeast Iran) and tectonic implications. Int J Earth Sci.

[CR2] Andersen DJ, Lindsley DH (1988). Internally consistent solution models for Fe–Mg–Mn–Ti oxides; Fe–Ti oxides. Amer Miner.

[CR3] Bachmann O, Dungan MA, Lipman PW (2002). The Fish Canyon Magma Body, San Juan Volcanic Field, Colorado: rejuvenation and Eruption of an Upper-Crustal Batholith. J Petrol.

[CR4] Bateman PC, Chappell BW (1979). Crystallization, fractionation, and solidification of the Tuolumne intrusive series, Yosemite National Park, California. Geol Soc Am Bull.

[CR5] Benisek A, Dachs E, Kroll H (2010). A ternary feldspar-mixing model based on calorimetric data: development and application. Contrib Mineral Petrol.

[CR6] Bindeman IN, Valley JW (2002). Oxygen isotope study of the Long Valley magma system, California: isotope thermometry and convection in large silicic magma bodies. Contrib Mineral Petrol.

[CR7] Blundy J, Wood B (1994). Prediction of crystal-melt partition coefficients from elastic moduli. Nature.

[CR8] Boehnke P, Watson EB, Trail D, Harrison TM, Schmitt AK (2013). Zircon saturation re-revisited. Chem Geol.

[CR9] Burke SR (2017) Zircon as proxy for “taking the temperature” of granites: an example using zircon thermometry applied to Greenvillian mid-crustal magmas in the Blue Ridge province, Virginia. Theses and Dissertations-Earth and Environmental Sciences, p 46

[CR10] Carley TL, Miller CF, Wooden JL, Padilla AJ, Schmitt AK, Economos RC, Bindeman NI, Jordan BT (2014). Iceland is not a magmatic analog for the Hadean: evidence from the zircon record. Earth Planet Sci Lett.

[CR11] Chamberlain KJ, Wilson CJ, Wooden JL, Charlier BL, Ireland TR (2013). New perspectives on the Bishop Tuff from zircon textures, ages and trace elements. J Petrol.

[CR12] Chappell BW, White AJR (1974). Two contrasting granite types. Pac Geol.

[CR13] Chappell BW, White AJR, Wyborn D (1987). The importance of residual source material (restite) in granite petrogenesis. J Petrol.

[CR14] Chappell B, Bryant C, Wyborn D, White A, Williams I (1998). High- and low-temperature I-type granites. Resour Geol.

[CR15] Chen L, Wang Z, Yan Z, Gong J, Ma S (2018). Zircon and cassiterite U–Pb ages, petro-geochemistry and metallogenesis of Sn deposits in the Sibao area, northern Guangxi: constraints on the neoproterozoic granitic magmatism and related Sn mineralization in the western Jiangnan Orogen, South China. Min Pet.

[CR16] Claiborne LL, Miller CF, Wooden JL (2010). Trace element composition of igneous zircon: a thermal and compositional record of the accumulation and evolution of a large silicic batholith, Spirit Mountain, Nevada. Contrib Mineral Petrol.

[CR17] Clemens J, Stevens G (2012). What controls chemical variation in granitic magmas?. Lithos.

[CR18] Clemens J, Vielzeuf D (1987). Constraints on melting and magma production in the crust. Earth Planet Sci Lett.

[CR19] Conolly JAD, Petrini K (2002). An automated strategy for calculation of phase diagram sections and retrieval of rock properties as a function of physical conditions. J Metamorph Petrol.

[CR20] Eby G. Nelson, Krueger Harold W., Creasy John W. (1992). Geology, geochronology, and geochemistry of the White Mountain batholith, New Hampshire. Geological Society of America Special Papers.

[CR21] Ferry JM, Watson EB (2007). New thermodynamic models and revised calibrations for the Ti-in-zircon and Zr-in-rutile thermometers. Contrib Mineral Petrol.

[CR22] Finger F, Clemens JD (1995). Migmatization and “secondary” granitic magmas: effects of emplacement and crystallization of “primary” granitoids in Southern Bohemia, Austria. Contrib Mineral Petrol.

[CR23] Finger F, Schiller D (2012). Lead contents of S-type granites and their petrogenetic significance. Contrib Mineral Petrol.

[CR24] Finger F, Steyrer HP (1988). Granite-types in the Hohe Tauern (Eastern Alps, Austria)–Some aspects on their correlation to Variscan plate-tectonic processes. Acta Geot.

[CR25] Frasl G, Finger F (1991). Geologisch-petrographische Exkursion in den österreichischen Teil des Südböhmischen Batholiths. Eur J Mineral.

[CR26] Frost BR, Barnes CG, Collins WJ, Arculus RJ, Ellis DJ, Frost CD (2001). A geo-chemical classification for granitic rocks. J Petrol.

[CR27] Fu B, Page FZ, Cavosie AJ, Fournelle J, Kita NT, Lackey JS, Wilde SA, Valley J (2008). Ti-in-zircon thermometry: applications and limitations. Contrib Mineral Petrol.

[CR28] Fuchs G, Thiele O (1968). Erläuterungen zur Übersichtskarte des Kristallins im westlichen Mühlviertel und im Sauwald, Oberösterreich: 1:100,000 (Ausgabejahr 1965).

[CR29] Gao P, Zheng YF, Zhao ZF (2016). Experimental melts from crustal rocks: a lithochemical constraint on granite petrogenesis. Lithos.

[CR30] Gervasoni F, Klemme S, Rocha-Júnior ER, Berndt J (2016). Zircon saturation in silicate melts: a new and improved model for aluminous and alkaline melts. Contrib Mineral Petrol.

[CR31] Ghent ED, Stout MZ (1984). TiO_2_ activity in metamorphosed pelitic and basic rocks: principles and applications to metamorphism in southeastern Canadian Cordillera. Contrib Mineral Petrol.

[CR32] Ghiorso MS, Gualda GAR (2012). A method for estimating the activity of titania in magmatic liquids from the compositions of coexisting rhombohedral and cubic iron-titanium oxides. Contrib Mineral Petrol.

[CR33] Grajales-Nishimura JM, Ramos-Arias MA, Solari L, Murillo-Muñetón G, Centeno-Garcia E, Schaaf P, Torres-Vargas R (2018). The Juchatengo complex: an upper-level ophiolite assemblage of late Paleozoic age in Oaxaca, southern Mexico. Int J Earth Sci.

[CR34] Gualda GAR, Ghiorso MS (2013). The Bishop Tuff giant magma body: an alternative to the Standard Model. Contrib Mineral Petrol.

[CR35] Gualda GA, Ghiorso MS, Lemons RV, Carley TL (2012). Rhyolite-MELTS: a modified calibration of MELTS optimized for silica-rich, fluid-bearing magmatic systems. J Petrol.

[CR36] Harrison TM, Schmitt AK (2007). High sensitivity mapping of Ti distributions in Hadean zircons. Earth Planet Sci Lett.

[CR37] Harrison TM, Watson EB, Aikman AB (2007). Temperature spectra of zircon crystallization in plutonic rocks. Geology.

[CR38] Hayden LA, Watson EB (2007). Rutile saturation in hydrous siliceous melts and its bearing on Ti-thermometry of quartz and zircon. Earth Planet Sci Lett.

[CR39] Hildreth W (1979). The Bishop Tuff: evidence for the origin of compositional zonation in silicic magma chambers. Spec Pap Geol Soc Am.

[CR40] Hildreth W, Wilson CJN (2007). Compositional zoning of the Bishop Tuff. J Petrol.

[CR41] Ickert R, Williams I, Wyborn D (2011). Ti in zircon from the Boggy Plain zoned pluton: implications for zircon petrology and Hadean tectonics. Contrib Mineral Petrol.

[CR42] Ishihara S (1977). The magnetite-series and ilmenite-series granitic rocks. Min Geol.

[CR43] Johannes W, Holtz F (1991). Formation and ascent of granitic magmas. Geol Rund.

[CR44] Kemp AIS, Whitehouse MJ, Hawkesworth CJ, Alarcon MK (2005). A zircon U–Pb study of metaluminous (I-type) granites of the Lachlan Fold Belt, southeastern Australia: implications for the high/low temperature classification and magma differentiation processes. Contrib Mineral Petrol.

[CR45] Kennedy A (2011). New U–Th–Pb isotope reference material for SIMS. Microsc Microanal.

[CR46] King P, White A, Chappell B, Allen C (1997). Characterization and origin of aluminous A-type granites from the Lachlan Fold Belt, southeastern Australia. J Petrol.

[CR47] Klomínský J, Jarchovský T, Rajpoot G (2010) Atlas of plutonic rocks and orthogneisses in the Bohemian Massif. Technical Report TR-01-2010, Czech Geologic Survey; Prague

[CR48] Langone A, Zanetti A, Daczko N, Piazolo S, Tiepolo M, Mazzucchelli M (2018). Zircon U–Pb dating of a lower crustal shear zone: a case study from the northern sector of the Ivrea-Verbano Zone (Val Cannobina, Italy). Tectonics.

[CR49] Liew TC, Finger F, Höck V (1989). The Moldanubian granitoid plutons of Austria: chemical and isotopic studies bearing on their environmental setting. Chem Geol.

[CR50] Middlemost EAK (1994). Naming materials in the magma/igneous rock system. Earth Sci Rev.

[CR51] Nabelek PI, Russ-Nabelek C, Denison JR (1992). The generation and crystallization conditions of the Proterozoic Harney Peak Leucogranite, Black Hills, South Dakota, USA: petrologic and geochemical constraints. Contrib Mineral Petrol.

[CR52] Powell R, Holland T (1999). Relating formulations of the thermodynamics of mineral solid solutions; activity modeling of pyroxenes, amphiboles, and micas. Am Miner.

[CR53] Reid MR, Vazquez JA, Schmitt AK (2011). Zircon-scale insights into the history of a Supervolcano, Bishop Tuff, Long Valley, California, with implications for the Ti-in-zircon geothermometer. Contrib Mineral Petrol.

[CR54] Rivera TA, Storey M, Schmitz MD, Crowley JL (2013). Age intercalibration of ^40^Ar/^39^Ar sanidine and chemically distinct U/Pb zircon populations from the Alder Creek Rhyolite Quaternary geochronology standard. Chem Geol.

[CR55] Roberts NM, Yang Q QY, Santosh M (2018). Rapid oxygen diffusion during high temperature alteration of zircon. Sci Rep.

[CR56] Ryerson FJ, Watson EB (1987). Rutile saturation in magmas: implications for Ti–Nb–Ta depletion in island-arc basalts. Earth Planet Sci Lett.

[CR57] Sepidbar F, Mirnejad H, Ma C (2018). Mineral chemistry and Ti in zircon thermometry: insights into magmatic evolution of the Sangan igneous rocks, NE Iran. Chem Erde.

[CR58] Shabanian N, Davoudian AR, Dong Y, Liu X (2017). U–Pb zircon dating, geochemistry and Sr–Nd–Pb isotopic ratios from Azna-Dorud Cadomian metagranites, Sanandaj-Sirjan Zone of Western Iran. Precambr Res.

[CR59] Siégel C, Bryan SE, Allen CM, Gust DA (2018). Use and abuse of zircon-based thermometers: a critical review and a recommended approach to identify antecrystic zircons. Earth Sci Rev.

[CR60] Steshenko E, Nikolaev A, Bayanova T, Drogobuzhskaya S, Chashchin V, Serov P, Lyalina L, Novikov A (2017). The paleoproterozoic Kandalaksha Anorthosite Massif: new U–Pb (ID-TIMS) data and geochemical features of zircon. Doklady Earth Sci.

[CR61] Stevens G, Villaros A, Jean-François Moyen JF (2007). Selective peritectic garnet entrainment as the origin of geochemical diversity in S-type granites. Geology.

[CR62] Thomas JB, Watson AB (2012). Application of the Ti-in-quartz thermobarometer to rutile-free systems. Reply to: a comment on: ‘TitaniQ under pressure: the effect of pressure and temperature on the solubility of Ti in quartz’ by Thomas et al.. Contrib Mineral Petrol.

[CR63] Vazquez JA, Kyriazis SF, Reid MR, Sehler RC, Ramos FC (2009). Thermochemical evolution of young rhyolites at Yellowstone: evidence for a cooling but periodically replenished postcaldera magma reservoir. J Volcanol Geotherm Res.

[CR64] Wark DA, Hildreth W, Spear FS, Cherniak DJ, Watson EB (2007). Pre-eruption recharge of the Bishop magma system. Geology.

[CR65] Watson EB (1996). Surface enrichment and trace-element uptake during crystal growth. Geoc Cosmoc.

[CR66] Watson EB, Harrison TM (1983). Zircon saturation revisited: temperature and composition effects in a variety of crustal magma types. Earth Planet Sci Lett.

[CR67] Watson EB, Liang Y (1995). A simple model for sector zoning in slowly grown crystals: for growth rate and lattice diffusion, with emphasis on accessory minerals in crustal rocks. Am Mineral.

[CR68] Watson EB, Wark DA, Thomas JB (2006). Crystallization thermometers for zircon and rutile. Contrib Mineral Petrol.

[CR69] Whalen JB, Currie KL, Chappell BW (1987). A-type granites: geochemical characteristics, discrimination and petrogenesis. Contrib Mineral Petrol.

[CR70] Whitney DL, Evans BW (2010). Abbreviations for names of rock-forming minerals. Am Miner.

[CR71] Williamson BJ, Shaw A, Downes H, Thirlwall MF (1996). Geochemical constraints on the genesis of Hercynian two-mica leucogranites from the Massif Central, France. Chem Geol.

[CR72] Wyborn D, Turner BS, Chappell BW (1987). The Boggy Plain Supersuite: a distinctive belt of I-type igneous rocks of potential economic significance in the Lachlan Fold Belt. Aust J Earth Sci.

[CR73] Žák J, Verner K, Janoušek V, Holub FV, Kachlík V, Finger F, Trubač J, Schulman K, Martínez-Catalán JR, Lardeaux JM, Janoušek V, Oggiano G (2014). A plate-kinematic model for the assembly of the Bohemian Massif constrained by structural relationships around granitoid plutons. The Variscan Orogeny: extent, timescale and the formation of the European Crust.

[CR74] Zhang Y, Shao Y, Liu Q, Chen H, Quan W, Sun A (2018). Jurassic magmatism and metallogeny in the eastern Qin-Hang Metallogenic Belt, SE China: an example from the Yongping Cu deposit. J Geochem Explor.

